# A multi-dentate, cooperative interaction between endo- and exo-ribonucleases within the bacterial RNA degradosome

**DOI:** 10.1093/nar/gkaf960

**Published:** 2025-10-02

**Authors:** Giulia Paris, Kai Katsuya-Gaviria, Hannah Clarke, Margaret Johncock, Tom Dendooven, Aleksei Lulla, Ben F Luisi

**Affiliations:** Department of Biochemistry, University of Cambridge, Tennis Court Road, Cambridge CB2 1GA, United Kingdom; Department of Biochemistry, University of Cambridge, Tennis Court Road, Cambridge CB2 1GA, United Kingdom; Department of Biochemistry, University of Cambridge, Tennis Court Road, Cambridge CB2 1GA, United Kingdom; Department of Biochemistry, University of Cambridge, Tennis Court Road, Cambridge CB2 1GA, United Kingdom; Division of Structural Studies, Laboratory of Molecular Biology, Francis Crick Avenue, Cambridge CB2 0QH, United Kingdom; Department of Biochemistry, University of Cambridge, Tennis Court Road, Cambridge CB2 1GA, United Kingdom; Department of Biochemistry, University of Cambridge, Tennis Court Road, Cambridge CB2 1GA, United Kingdom

## Abstract

In *Escherichia coli* and numerous other bacteria, two of the principal enzymes mediating messenger RNA decay and RNA processing—RNase E, an endoribonuclease, and polynucleotide phosphorylase (PNPase), an exoribonuclease—assemble into a multi-enzyme complex known as the RNA degradosome. While RNase E forms a homotetramer and PNPase a homotrimer, it remains unclear how these two enzymes interact within the RNA degradosome to potentially satisfy all mutual recognition sites. In this study, we used cryo-EM, biochemistry, and biophysical studies to discover and characterize a new binding mode for PNPase encompassing two or more motifs that are necessary and sufficient for strong interaction with RNase E. While a similar interaction is seen in *Salmonella enterica*, a different recognition mode arose for *Pseudomonas aeruginosa*, illustrating the evolutionary drive to maintain physical association of the two ribonucleases. The data presented here suggest a model for the quaternary organization of the RNA degradosome of *E. coli*, where one PNPase trimer interacts with one RNase E protomer. Conformational transitions are predicted to facilitate substrate capture and transfer to catalytic centres. The model suggests how the endo- and exo-ribonucleases might cooperate in cellular RNA turnover and recruitment of regulatory RNA by the degradosome assembly.

## Graphical Abstract

**Graphical Abstract:**
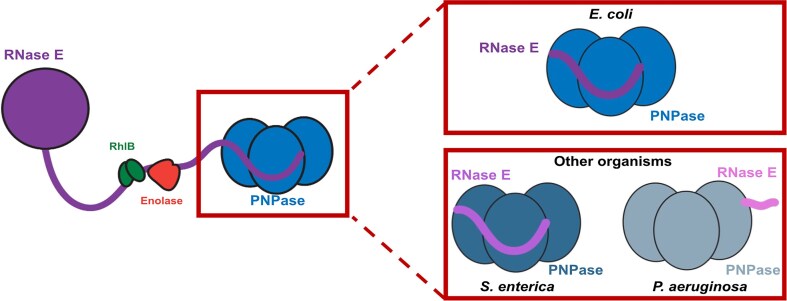

## Introduction

One key process underpinning the phenotypic complexity of all life forms is the turnover of RNA. In diverse organisms, the enzymes involved in RNA metabolism often form complex hetero-oligomeric assemblies that enrich regulatory capacity. For example, some of these nanomachines act to intercept transcripts tagged with complementary regulatory RNAs for rapid, targeted turnover. This process helps to fine-tune gene expression and can generate elaborate networks linking different gene expression pathways. Many assemblies that function in transcript turnover also serve to mature intermediates of structural RNA species. The multi-enzyme assemblies are, in some cases, compartmentalized [1] or form nano-organelles through condensation [2–6], with impact on temporal and spatial organization of RNA metabolism [3, 5]. Diverse in composition and organization across phyla, these machines have in many cases arisen by evolutionary convergence, implicating the biological utility of forming multi-functional assemblies to meet the requirements for RNA metabolism in diverse life forms.

**Figure 1.**
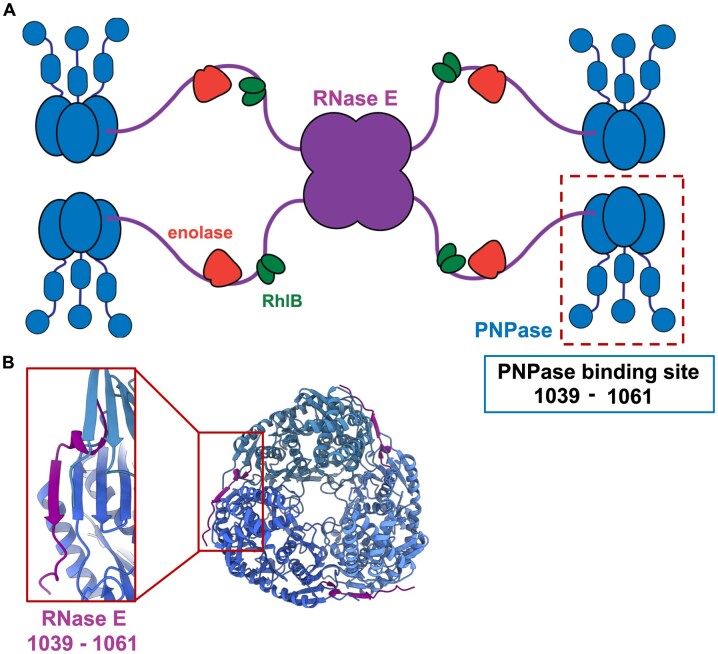
Schematic of model for the multi-enzyme RNA degradosome. (**A**) Schematic of the RNA degradosome in which a RNase E tetramer (purple) interacts with the canonical components of the complex, namely PNPase (blue), enolase (orange), and the ATP-dependent DEAD RNA helicase RhlB (green). In this schematic, each RNase E protomer interacts with one PNPase trimer. The PNPase recognition microdomain, identified by previous studies [7, 19], is indicated in the blue box. In this study, a second binding site is identified that boosts overall affinity. (**B**) The interaction of the RNase E microdomain (1039–1061) with PNPase core, elucidated by *X*-ray crystallography (PDB: 3GCM) [19] and corroborated here by cryo-EM. Some residues of the RNase E microdomain form an antiparallel β-strand with the β-sheet part of the C-terminal RNase PH-like subdomain of PNPase core.

In the model gram-negative bacterium *Escherichia coli*, a multi-enzyme assembly, referred to as the RNA degradosome, plays a central role in RNA metabolism [7]. At the core of the assembly is the endoribonuclease RNase E, which initiates turnover of most messenger RNA (mRNA) transcripts and processes precursors of ribosomal RNAs, transfer RNAs, and small regulatory RNAs (sRNAs) [8, 9]. Once a transcript is cleaved by RNase E, the RNA is often committed to a fate of rapid degradation. Beyond the residues that form the catalytic core (1–529), each protomer of RNase E presents an intrinsically disordered C-terminal domain (CTD), extending from residues 530–1061, that serves as a scaffold for the RNA degradosome [10, 11]. The activity and functionality of RNase E is increased by its assembly into the RNA degradosome [12]. While the composition of the RNA degradosome can change with growth conditions, its canonical partners are the ATP-dependent DEAD-box helicase RhlB, the glycolytic enzyme enolase, and the exoribonuclease polynucleotide phosphorylase (PNPase) (Fig. 1A) [13]. PNPase was originally discovered as a polynucleotide or poly(A) polymerase in bacteria such as *E. coli*. Notwithstanding, PNPase predominantly acts as phosphorolytic 3′ → 5′ exoribonuclease that catalyses the processive degradation of single-stranded RNA, involved both in mRNA decay and sRNA-mediated gene regulation [14, 15]. Moreover, the RNA degradosome can recruit the RNA chaperone Hfq and sRNAs, providing several potential binding sites for the Hfq/sRNA complexes [16]. One of the potential binding partners for the Hfq/sRNA complexes is PNPase, and the formation of this ternary complex was shown to protect the sRNA against ribonucleases [17]. The intrinsically disordered nature of the scaffold domain of the RNA degradosome is a conserved feature [18] that has been associated with the propensity to form microscopic condensates *in vivo* [1, 6] and *in vitro* [4]. Condensate formation may favour cooperation of the activities of the degradosome.

Predictions and experimental studies of the RNA degradosome and its subassemblies have identified binding motifs for the canonical components on the scaffold domain of RNase E (Fig. 1A). A conserved motif mediates a weak interaction with PNPase via the formation of a β-strand that extends an exposed β-sheet of the PNPase core (Fig. 1B) [19], with a measured dissociation constant in the micromolar range [19, 20]. However, such a comparatively weak affinity raises the question of how the two key ribonuclease components interact avidly *in vivo*. In addition, with RNase E forming a homo-tetramer and PNPase a homo-trimer, it is unclear how the respective interaction sites are matched so that all are occupied. One scenario is that different RNA degradosome molecules could cluster together and be bridged by sharing PNPase trimers. Another possibility is for three RNase E CTDs belonging to the same RNase E homotetramer to interact with one PNPase trimer [21]. Here, we present experimental evidence from cryo-EM and biophysical studies revealing a third scenario, where each protomer of RNase E recruits one PNPase trimer through the interaction with two PNPase protomers, increasing the catalytic activity of PNPase. We propose that the same PNPase trimer may form transient interactions with a third distal motif in RNase E that could facilitate substrate capture and delivery to active sites, impacting the activity of the two ribonucleases in the cellular context.

## Materials and methods

### Multiple sequence alignments

The *E. coli* RNase E sequence was used as a reference for a BLASTP search of homologs (BLOSUM62) [22]. A subset of these sequences was selected based on previous literature [18] for a multiple sequence alignment using Clustal Omega [23]. The multiple sequence alignment was visualized using Jalview [24].

### Expression vector cloning and purification of proteins

#### PNPase and PNPase–RNase E co-purified complexes

RNase E constructs encoding for RNase E 1000–1061 (used for degradation assays) and RNase E 960–1061 (used for cryo-EM) were amplified by PCR. PNPase, and the co-expressed PNPase–RNase E 1000–1061 and PNPase–RNase E 960–1061 complexes were expressed and purified following the same protocol presented in Dendooven *et al.* (2021) [17] and summarized here. Transformed *E. coli* BL21(DE3) cells were grown in 2×YT media at 37°C and induced at early exponential phase with 0.5 mM isopropyl-β-D-thiogalactopyranoside (IPTG), following which the temperature was reduced to 25°C. Four hours post-induction, cells were harvested by centrifugation, suspended in Lysis Buffer 1 [20 mM Tris–HCl, pH 8.0, 150 mM NaCl, 150 mM KCl, 5 mM MgCl_2_, 1 mM Ethylenediaminetetraacetic acid (EDTA)], lysed using a cell disruptor (Emulsiflex, Avestin), and the lysate was clarified by centrifugation (4°C, 30 min, 37 500 × *g*). Ammonium sulfate was added to the supernatant to 51.3% saturation. Following centrifugation (4°C, 30 min, 37 500 × *g*), the pellet was resuspended in Q Buffer A′ [20 mM Tris–HCl, pH 8.5, 0.5 mM tris(2-carboxyethyl)phosphine (TCEP), 10% v/v glycerol, EDTA-free protease inhibitor cocktail (Roche)] to solubilize PNPase. The solubilized sample was loaded onto a 5 ml HiTrap Q column (GE Healthcare), equilibrated in Q Buffer A (20 mM Tris–HCl, pH 8.5, 30 mM NaCl, 0.5 mM TCEP, 10% v/v glycerol), and eluted with a gradient of Q Buffer B (20 mM Tris–HCl, pH 8.5, 1 M NaCl, 0.5 mM TCEP, 10% v/v glycerol). Fractions containing PNPase were pooled, mixed with an equal volume of Supplementation Buffer [1 mM MgCl_2_, 45 mM NaH_2_PO_4_, pH 7.9, 0.9 M (NH_4_)_2_SO_4_, 1 mM TCEP], and loaded on a 5 ml HiTrap butyl-Sepharose HIC column (GE Healthcare) equilibrated with BS Buffer A [50 mM Tris–HCl, pH 7.5, 1 M (NH_4_)_2_SO_4_, 0.5 mM TCEP], and eluted with a gradient of BS Buffer B (50 mM Tris–HCl, pH 7.5). Fractions enriched in PNPase were pooled, concentrated, and loaded on a Superdex 200 Increase 10/300 gel filtration column equilibrated with PNPase Storage Buffer [20 mM Tris–HCl, pH 8.0, 150 mM NaCl, 5 mM MgCl_2_, 0.5 mM TCEP, 10% (v/v) glycerol].

#### Affinity tagged RNase E C-terminal domain segments

RNase E genes encoding for the different RNase E fragments were amplified by PCR and cloned into a pMxe plasmid, which presents several tags at the N-terminal and C-terminal regions. Briefly, at the N-terminal of the construct, there is an Hisx6 affinity tag that will be used for affinity purification. Following the Hisx6 tag there is an maltose-binding protein (MBP) tag with a TEV cleavage site to maintain solubility of the peptides [25]. Following the MBP tag the gene encoding the peptide is inserted, and is then followed by intein, a self-cleavable tag, followed by a choline-binding domain, which can be used for an extra step of affinity purification [26]. RNase E segments were expressed from a pMxe vector in *E. coli* Evo21(DE3) [27] cells induced with 0.4 mM IPTG at 18°C overnight. After expression, cells were harvested, resuspended in Lysis Buffer 2 (20 mM Tris–HCl, pH 8.0, 80 mM KCl, 300 mM NaCl, 20 mM Imidazole, 0.5 mM TCEP), and lysed with a cell disruptor (Emulsiflex, Avestin). The lysate was clarified by centrifugation (4°C, 30 min, 37 500 × *g*), and the clarified supernatant was applied to a Ni-NTA gravity flow column, equilibrated with Ni Equilibration Buffer (20 mM Tris–HCl, pH 8.0, 300 mM NaCl, 20 mM imidazole, 0.5 mM TCEP). After subsequent rounds of washing with Ni Equilibration Buffer supplemented with, respectively, 0.1% Triton-X, 1 M NaCl, and a final wash in Ni Equilibration Buffer, samples were eluted with Ni Elution Buffer (20 mM Tris–HCl, pH 8.0, 300 mM NaCl, 300 mM imidazole, 0.5 mM TCEP). Enriched fractions were then supplemented with 100 mM β-mercaptoethanol to catalyse intein self-cleavage overnight at room temperature. Treated samples were filtered and loaded on an amylose gravity flow column, equilibrated with Amy Equilibration Buffer (20 mM Tris–HCl, pH 8.0, 20 mM NaCl, 20 mM imidazole, 2 mM β-mercaptoethanol) and after washing, His-tagged TEV protease was applied to the resin and incubated at 4°C for 2 h. The cleaved product was then loaded on a Ni-NTA gravity flow column, equilibrated with Ni Equilibration Buffer (20 mM Tris–HCl, pH 8.0, 300 mM NaCl, 20 mM imidazole, 0.5 mM TCEP), to separate the TEV protease and the His-MBP tag from the RNase E peptide. The flow-through from Ni-NTA was then loaded on a 5 ml HiTrap Q column (GE Healthcare), equilibrated in Q Load Buffer (20 mM Tris–HCl, pH 8.0, 20 mM NaCl, 0.5 mM TCEP) and eluted with a linear gradient 0–100% of Q Elution Buffer (20 mM Tris-HCl pH 8.0, 1 M NaCl, 0.5 mM TCEP). The peak containing the RNase E segments of interest was then concentrated and loaded on a Superdex 75 10/300 GL gel filtration column equilibrated with SPR Buffer (20 mM HEPES, pH 7.2, 150 mM NaCl, 5 mM MgCl_2_, 0.5 mM TCEP).

#### Purification of PNPase core

The PNPase core encoding sequence from *E. coli* (aa 1–552), *Salmonella enterica* serovar Typhimurium (UniProt: Q8ZLT3) (aa 1–552), or *Pseudomonas aeruginosa* (strain PAO1, UniProt: Q9HV59) (aa 1–553) was amplified using corresponding genomic DNA as a template and cloned into pExp-Mxe-CHis expression vector. The protein of interest, produced as a fusion with C-terminal 8×His-tagged *Mycobacterium xenopi* GyrA intein, was expressed in Evo21(DE3) cells at 18°C overnight following induction with 0.4 mM IPTG. The proteins were purified first by immobilized metal affinity chromatography (IMAC) using Ni-NTA resin (Qiagen), then 50 mM β-mercaptoethanol was added to Ni-eluate to activate intein cleavage for 16 h at 18°C. After cleavage, the protein buffer was exchanged using HiPrep 26/10 desalting column (Cytiva), and the protein solution was reapplied to Ni-NTA resin to remove His-tagged intein and uncleaved material. The protein was then purified by hydrophobic interaction chromatography using HiTrap Butyl HP column (Cytiva), anion-exchange chromatography using HiTrap Q HP column (Cytiva) and, finally, by size-exclusion chromatography using Superdex 200 Increase 10/300 GL column (Cytiva). After the last step the protein solution in GF buffer (20 mM HEPES-NaOH pH 7.2, 200 mM NaCl, 5 mM MgCl_2_, 0.5 mM TCEP) was concentrated to 10 mg/ml using Amicon Ultra concentrator with 30 kDa cut-off (Millipore).

#### Purification of RNase E-muGFP-Chis

The DNA corresponding to RNase E aa residues 921–1061 in *E. coli*, aa 921–1067 in *S. enterica* or aa 883–1057 in *P. aeruginosa* was amplified from corresponding genomic DNA and cloned into pExp-MBP-TEV-muGFP-CHis vector. The fusion protein, where sequence of interest was sandwiched between N-terminal MBP followed by TEV protease cleavage site and C-terminal 8×His-tagged muGFP [28], was produced in Evo21(DE3) cells after overnight induction with 0.4 mM IPTG at 18°C. The protein was initially purified by IMAC using Ni-NTA resin (Qiagen) and then by affinity chromatography using amylose resin (NEB). The MBP-fusion tag was cleaved by treatment with TEV protease (produced in-house) for 16 h at 18°C. The protein was further purified by anion-exchange chromatography using HiTrap Q HP column (Cytiva) and then eluted from size-exclusion chromatography using Superdex 200 Increase 10/300 GL column (Cytiva) equilibrated in GF buffer.

#### PNPase C-His tag

PNPase carrying a C-terminal Hisx6 tag was expressed from pExp-*cis-*Ec_PNP-CHis vector in *E. coli* Evo21(DE3) cells, induced with 0.4 mM IPTG, at 18°C overnight. After expression, cells were harvested and resuspended in Lysis Buffer 2 (20 mM Tris–HCl, pH 8.0, 80 mM KCl, 300 mM NaCl, 20 mM imidazole, 0.5 mM TCEP), and lysed with a cell disruptor (Emulsiflex, Avestin). Subsequently, the lysate was clarified by centrifugation (4°C, 30 min, 37 500 × *g*), and the clarified supernatant was filtered and applied to a Ni-NTA gravity flow column equilibrated with Ni PNPCHis Equilibration Buffer (20 mM NaH_2_PO_4_, pH 8.0, 300 mM NaCl, 300 mM KCl, 5 mM MgCl_2_, 20 mM imidazole, pH 7.5, 10 mM β-mercaptoethanol). After subsequent rounds of washing with equilibration buffer supplemented with 0.1% Triton-X, or 1 M NaCl, the sample was eluted with Ni PNPCHis Elution Buffer (20 mM NaH_2_PO_4_, pH 8.0, 300 mM NaCl, 300 mM KCl, 5 mM MgCl_2_, 300 mM imidazole, pH 7.5, 10 mM β-mercaptoethanol). The eluted PNPase C-His was supplemented with Supplementation Buffer [1 mM MgCl_2_, 45 mM NaH_2_PO_4_, pH 7.9, 0.9 M (NH_4_)_2_SO_4_, 1 mM TCEP] and loaded on a 5 ml HiTrap butyl-sepharose HIC column (GE Healthcare) equilibrated with Butyl PNPCHis A [20 mM NaH_2_PO_4_, pH 8.0, 1 M (NH_4_)_2_SO_4_, 0.5 mM TCEP] and eluted with a gradient of Butyl PNPCHis B (20 mM NaH_2_PO_4_, pH 8.0). Fractions containing PNPase were run through a HiPrep™ 26/10 Desalting Column (GE Healthcare) and buffer exchanged to Q PNPCHis A (20 mM NaH_2_PO_4_, pH 8.0, 30 mM NaCl, 10% glycerol, 0.5 mM TCEP). The buffer-exchanged sample was then loaded on a 5 ml HiTrap Q column (GE Healthcare), equilibrated in Q PNPChis A and eluted with a linear gradient 0%–100% of Q PNPCHis B (20 mM NaH_2_PO_4_, pH 8.0, 1 M NaCl, 10% glycerol, 0.5 mM TCEP). Fractions enriched with PNPase C-His from the 5 ml Q column were combined, concentrated and loaded on a Superdex S200 10/300 GL gel filtration column equilibrated with SPR Buffer (20 mM HEPES, pH 7.2, 150 mM NaCl, 5 mM MgCl_2_, 0.5 mM TCEP).

## Cryo-EM

### Grid preparation and cryo-EM data collection

Purified PNPase core and RNE-muGFP-CHis proteins were mixed in 1:2 ratio and their complex were separated on the Superdex 200 Increase 10/300 GL column (Cytiva) equilibrated with cryo-EM Buffer (20 mM Tris–HCl, pH 8.0, 25 mM MgCl_2_, 150 mM KCl, 1 mM TCEP). Peak fractions were combined, the protein was concentrated to 15 μM using Amicon Ultra concentrator with 10 kDa cut-off (Millipore) and used to prepare cryo-EM grids. The samples were mixed with CHAPSO (3-([3-cholamidopropyl]dimethylammonio)-2-hydroxy-1-propanesulfonate) at a final concentration of 8 mM before being applied onto R2/2 Au Ultrafoil grids (Quantifoil) that had been pre-treated by glow-discharge (PELCO easiGLOW: 60 s, 25 mA, 0.39 mBar). The grids were blotted, and sample vitrified in liquid ethane using a FEI Vitrobot (IV) (100% humidity, 4°C, blotting force -4, 3 sec blot time). Grids were pre-screened on a FEI Talos Arctica and high-resolution datasets were collected on a FEI Titan Krios equipped with a Falcon 4i-Selectris camera and energy filter. The data collection parameters for all specimens are summarized in Table 2.

### Cryo-EM data processing of *E. coli* PNPase:RNase E 921-1061-muGFP

Six thousand one hundred twelve movies were collected on a FEI Titan Krios with a Falcon 4i Selectris detector and processed in cryoSPARC v 4.5.3 [29]. Following Patch Motion Correction and Patch CTF Estimation, particles were picked first using Blob Picker, then good 2D templates were used for a round template picking. 515k particles were picked and run through 2D classification. A summary of the data processing of the dataset can be found in Supplementary Fig. S3. Classes showing PNPase in different orientations were used to generate three *ab initio* models, and heterogeneous refinement was subsequently used to further improve the particle set. The best class (259k particles) was further classified by rounds of *ab initio* reconstruction and heterogeneous refinement. Heterogeneous and homogeneous refinements confirmed the presence of only one RNase E–GFP peptide bound to a PNPase trimer, and subsequently the density corresponding to GFP was masked out by focused 3D refinement to improve the resolution. The overall resolution of the cryo-EM map is estimated to be 2.52 Å (Supplementary Fig. S3).

### Cryo-EM data processing of *S. enterica* PNPase:RNase E 921-1061- muGFP

Four thousand movies were collected on a FEI Titan Krios with a Falcon 4i Selectris detector. Motion correction and CTF estimation were carried out in Warp 0.9 [30]. Particles were picked and extracted in Warp and subsequently imported in cryoSPARC v 4.5.3 where all the data processing was carried out [29]. Classes presenting PNPase in different orientations were used to generate three *ab initio* models. Heterogeneous refinement was subsequently used to further improve the particle set. Homogeneous refinement confirmed the presence of only one RNase E–GFP peptide bound to a PNPase trimer, and subsequently the density corresponding to GFP was masked out by focused 3D refinement. The overall resolution of the cryo-EM map is estimated to be 2.42 Å (Supplementary Fig. S3).

### Cryo-EM data processing of *P. aeruginosa* PNPase:MBP-RNase E 833-1067

Twelve thousand one hundred forty-seven movies were collected on a FEI Titan Krios with a Falcon 4i Selectris detector and processed in cryoSPARC v 4.6.2 [29]. Following Patch Motion Correction and Patch CTF Estimation, exposures were manually curated excluding those with poor CTF fit. 672k particles were picked using Blob Picker and run through 2D classification. Particles from classes showing clear PNPase density were used to generate *ab initio* models, and heterogeneous refinement was subsequently used to further improve the particle set. *Ab initio* reconstruction and heterogeneous refinement were used iteratively to further classify the particle set. A set of 204k particles showing density for RNase E bound to PNPase was refined and subsequently a mask focused on the RNase E peptide was used for focused 3D Classification. Following focused 3D classification, 41k particles showed an enhanced density corresponding to the RNase E peptide, and several rounds of refinement generated a map with overall resolution of 2.6 Å (Supplementary Fig. S3).

### Model building

Atomic models for the three complexes analysed in this study were built with the automated fitting program ModelAngelo [31]. Subsequently, all models were manually inspected and adjusted in Coot (version 0.9.8.95) [32]. Finally, all models were refined using Phenix 1–21 [33, 34].

### Surface plasmon resonance

Interaction measurements were made using a Biacore T200 instrument (GE) at 25°C. PNPase with a 6xHisTag at the CTD was immobilized on a Series S Sensor Chip NTA (Cytiva) to provide a signal of 5 response units (RU). Binding experiments were performed in SPR Buffer (20 mM HEPES, pH 7.2, 150 mM NaCl, 5 mM MgCl_2_, 0.5 mM TCEP) supplemented with 0.05% v/v Tween 20. Binding to PNPase was determined at six different concentrations (0, 111, 333, 1000, 3000, and 9000 nM for RNase E 960–1038, RNase E 1039–1061, and RNase E 960–1053, and 0, 5, 50, 500, 1000, and 5000 nM for RNase E 985–1061). For the SPR analysis of the point mutants, binding to PNPase was measured at four different concentrations (0, 12.5, 25, and 50 nM). All concentrations were run in duplicates. The contact time was 60 s, while the dissociation time was 120 s, at a 30 ml/min flow rate. The contact time for RNase E 960–1061, and all the point mutants was increased to 120 s. The chip surface was regenerated with 350 mM EDTA in water, following manufacturer's instructions. Data were processed using the manufacturer’s BIAevaluation software.

### Size-exclusion chromatography with multi-angle laser light scattering

Estimation of molar masses of PNPase and the various PNPase/RNase E complexes was carried out by size-exclusion chromatography with multi-angle laser light scattering (SEC-MALLS). Proteins (100 μl at ∼1 mg/ml concentration) were loaded onto a Superdex 200 10/300 Increase GL SEC column (GE Healthcare) at 0.5 ml/min using an AKTA Purifier FPLC system (GE Healthcare) in SEC-MALLS buffer (50 mM Tris–Cl, pH 7.5, 100 mM NaCl, 100 mM KCl, 1 mM TCEP). Chromatograms were acquired using UNICORN software (GE Healthcare) at 280 nm. The eluent from the S200 column was analysed with an online DAWN HELEOS II MALLS instrument with eight detectors (Wyatt Technology), followed by an Optilab T-rEX differential refractometer (Wyatt Technology) at 25°C. Data collection and analysis were performed using ASTRA 6 software (Wyatt Technology). Eluted peaks from SEC column were evaluated by Zimm Plot, using a dn/dc value of 0.1850 ml/g. Bovine serum albumin was used as a standard to confirm the system performance (Supplementary Table S1).

### *In vitro* transcription of RNA

The RNAs RyhB, CyaR, MicA, and GcvB were produced by *in vitro* transcription (IVT), using DNA oligos as template. Double-stranded DNA templates incorporating an upstream T7 promoter were prepared by annealing complementary oligonucleotides. Briefly, 20 μM of each oligo were mixed in RNase-free water and annealed by incubating for 15 min at 95°C allowing to cool to a temperature of 40°C. The oligo-mix was then used as a template for the IVT reaction. 200 µL IVT mixtures containing 3.5 μg of template DNA, T7 RNA polymerase, 5 mM of each of the rNTPs, 10 mM dithiothreitol (DTT), 0.5 U/μl RNaseOUT™ Recombinant Ribonuclease Inhibitor (Invitrogen) and 3% v/v dimethyl sulfoxide (DMSO) were incubated in IVT reaction Buffer (40 mM Tris, pH 8, 25 mM MgCl_2_, 2 mM spermidine) at 37°C for 5 h. IVT products were incubated with Turbo DNase I (Invitrogen) at 37°C for 30 min. DNase treated samples were incubated at room temperature with 50 mM EDTA for 5 min. RNA was purified using PureLink™ RNA Micro Kit (Invitrogen) columns and its integrity was verified by denaturing urea-PAGE. Purified RNA was stored in nuclease-free water at −20°C.

### RNA degradation assays

Degradation assays were performed using RyhB, CyaR, MicA, and GcvB RNAs as substrates for free PNPase or PNPase bound to RNase E (1000–1061). RNA was annealed by incubating for 2 min at 50°C and letting cool to room temperature. Degradation reactions were initiated by adding either free PNPase or the PNPase-RNase E (1000–1061) complex to a final concentration of 50 nM into a solution containing 500 nM of RNA in PNPase degradation buffer (20 mM Tris pH 7.5, 100 mM NaCl, 1 mM DTT, 1 mM MgCl_2_, 2 mM sodium phosphate) pre-incubated at 37°C. A time point (t=0) was taken immediately before enzyme addition. The reactions were carried out at 37°C and stopped at the indicated time points (1, 5, and 15 min) by adding an equal volume of 0.5 mg/ml Proteinase K in Proteinase K buffer (100 mM Tris–HCl, pH 7.5, 150 mM NaCl, 12.5 mM EDTA, and 1% w/v sodium dodecyl sulphate) and incubated at 50°C for 30 min. Reaction products from each timepoint were resolved on a 10% denaturing polyacrylamide gel (7.5 M urea) in 1×Tris/Borate/EDTA (TBE), stained with SYBR Gold solution (Invitrogen) and visualized by fluorescence imaging. Band intensities were quantified using ImageJ.

## Results

### RNase E C-terminal domain presents an extended PNPase binding site

**Figure 2.**
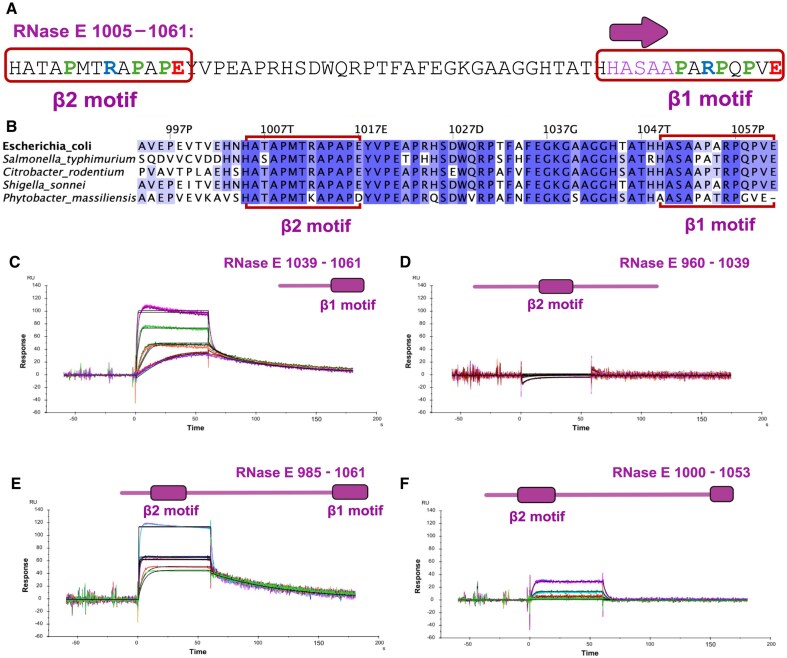
RNase E interacts with PNPase through a highly conserved binding motif. (**A**) Sequence of *E. coli* RNase E from residues 1005–1061. Similar residues to those that form the PNPase binding motif (β1 motif, residues 1049–1061) can be found from residues 1005–1017 (β2 motif), with a similar pattern of prolines (in green) and charged amino acids (Arg, in blue; Glu, in red). Residues coloured in purple are those involved in the formation of a β-strand upon interaction with PNPase [19]. (**B**) Sequence alignment of RNase E homologs, highlighting the characterized PNPase-interacting microdomain at the C-terminus (boxed in red) and the newly predicted site upstream (also boxed in red). Residues are shaded in blue by Percentage Identity, with greatest intensity corresponding to 100%. (**C**) SPR analysis of the interaction between PNPase and the RNase E peptide 1039–1061 resulted in the micromolar range, confirming what was previously described in the literature [19] (Table 1). (**D**) SPR analysis of the interaction between PNPase and the RNase E peptide 960–1039, carrying the β2 motif, suggests that the peptide does not interact with PNPase. (**E**) SPR analysis of the interaction between PNPase and the RNase E peptide 985–1061. The presence of the β2 motif, in addition to the β1 motif, boosts RNase E binding affinity to PNPase from the micromolar range to the low nanomolar range. (**F**) SPR analysis of the interaction between PNPase and the RNase E peptide 985–1053 revealed that in the absence of the conserved proline motif between residues 1053 and residues 1061 the binding affinity to PNPase decreases from the low nanomolar range to high micromolar.

Previous studies identified a PNPase binding motif encompassing residues 1039–1061 at the very C-terminus of *E. coli* RNase E (Fig. 1) [7]. A crystal structure of a peptide encompassing RNase E residues 1021–1061 bound to PNPase revealed that a short segment of RNase E (1048–1053) forms a β-strand that extends an exposed β-sheet of the PNPase core (Fig. 1B) [19]. Following the β-strand, there is a stretch of eight residues containing three prolines and two charged amino acids. The RNase E residues 1048–1061 will be referred to as ‘β1 motif’ hereafter (Fig. 2A). Multiple sequence alignment shows that the β1 motif is strongly conserved in RNase E among different bacteria, and the conservation of the motif has been reported in previous studies [18]. Additionally, the motif 1009–1017 was identified as a plausible secondary PNPase binding site and observed to be present in RNase E homologs in *Vibrionales*, *Pasteurellales*, and *Enterobacteriales* [18]. Comparing residues 1005–1017 with the β1 motif, a similar sequence pattern is observed, involving His residues followed by prolines and charged residues (Fig. 2A). Moreover, we observed that the more extended RNase E 1000–1061 segment is strongly conserved in other bacteria (Fig. 2B). For clarity, the region 1005–1017 of RNase E will be called ‘β2 motif’ hereafter (Fig. 2A). Generally, regions of intrinsic disorder bear little sequence conservation, but the punctuation of such regions by a small segment of conservation such as β2 motif is a hallmark of a short linear recognition motif [35]. Therefore, we speculated that the extended conserved region (1005–1061), encompassing both β1 motif and β2 motif, could have a structural and/or functional role in the binding of RNase E to PNPase.

The binding behaviour of RNase E segment 1005–1061 to PNPase was measured by Surface Plasmon Resonance (SPR) (Fig. 2C–F). First, the binding of the RNase E peptide carrying the previously described binding site (residues 1039–1061) to PNPase, resulted in a *K*_D_ in the low μM range, in agreement with previously reported observations from isothermal titration calorimetry affinity [19] (Table 1 Fig. 2C). Secondly, the binding of RNase E 960–1039 to PNPase, carrying the plausible secondary binding motif β2, was tested, but no binding was detected (Fig. 2D). When the peptide included both motifs (residues 985–1061), it exhibited a *K*_D_ in the low nanomolar range for binding to PNPase (Table 1 and Fig. 2E). The SPR data suggest that the newly identified motif, while unable to bind measurably to PNPase by itself under the conditions tested, strongly boosts the affinity of RNase E for PNPase when present together with the primary binding site. The increase in affinity is due to both acceleration of on-rate and decrease of off-rate (Table 1). Taken together, these observations support the hypothesis that the PNPase binding region of RNase E encompasses residues 1005–1061 of RNase E, rather than just the minimal 1039–1061 segment that was previously described.

**Table 1.** Rate constants and binding strengths of interactions between RNase E peptides and PNPase

Peptide	*k*_a_ (1/M s)	*k*_d_ (1/s)	*K*_D_ (M)
RNase E 1039-1061	1.40E + 5	0.833	5.92E-6
RNase E 985-1061	3.93E + 6	0.0158	4.02E-9
RNase E 985-1053	2.94E + 4	0.339	1.15E-5

The binding profiles are shown in Fig. 1, and a detailed summary of all peptides used in this study is presented in Supplementary Table S1.

**Table 2.** Cryo-EM data collection parameters

Structure	*E. coli* PNPase core:RNase E 921–1061-muGFP	*S. enterica* PNPase core:RNase E 921–1061-muGFP	*P. aeuroginosa PNPase core:MBP-RNase E 930 - 1051*
Data collection			
Microscope	Titan Krios G2	Titan Krios G2	Titan Krios G2
Voltage (kV)	300	300	300
Detector	Falcon 4i Selectris	Falcon 4i Selectris	Falcon 4i Selectris
Nominal magnification	165 000×	165 000×	165 000×
Pixel size (Å)	0.729	0.729	0.729
Electron dose, per frame (e-, Å^2^/fraction)	1.02	1.08	1.01
Electron dose, total (e-, Å^2^)	51.05	53.94	50.47
Defocus range (um)	−2.2, −2.0, −1.8, −1.6, −1.4, −1.2, −1.0, −0.8	−1.8, −1.6, −1.4, −1.2, −1.0, −0.8, −0.6	−1.8, −1.6, −1.4, −1.2, −1.0, −0.8, −0.6
Exposure (s)	4.39	4.39	4.39
Frames	50	50	50
Number of micrographs	6112	4000	12 147
Reconstruction			
Software	cryoSPARC v 4.6.2	cryoSPARC v 4.5.3	cryoSPARC v 4.6.2
Number of particles used	108 374	95 237	41 901
Final resolution (Å)	2.52	2.42	2.40
Symmetry	C1	C1	C1

Although not engaged in β-strand formation upon PNPase binding [19], RNase E residues 1053–1061 are strongly conserved (Fig. 2B). These residues show a high content in proline punctuated with charged residues, and the same pattern is seen from residues 1009–1017. To explore the role of the terminal proline-rich region, the binding to PNPase of an RNase E peptide lacking the terminal Pro-rich motif (residues 985–1053) was also tested by SPR (Table 1 and Fig. 2F). This version of RNase E peptide bound PNPase with a *K*_D_ of 100 μM, an affinity 10^4^ times lower compared to the full-length binding site, indicating that the proline motif is an important determinant of the interaction.

### Cryo-EM reveals an extended PNPase binding site on RNase E

To structurally characterize the newly identified extended PNPase binding region, an RNase E peptide (960–1061) was co-purified with PNPase and the complex was studied by cryo-EM. The preliminary cryo-EM map obtained using full-length PNPase showed that the RNase E peptide does not interact with the KH and S1 domains of PNPase (Supplementary Fig. S1), and to improve the overall reconstruction and reduce flexibility, a truncated version of PNPase, the PNPase core (1–550) lacking the KH and S1 domains, was used for structural analysis. The refined cryo-EM map of the purified sample revealed density corresponding to the RNase E peptide interacting with two protomers of the trimeric PNPase core (Supplementary Fig. S1). However, the resolution of the RNase E peptide was insufficient to allow confident alignment of the sequence.

**Figure 3.**
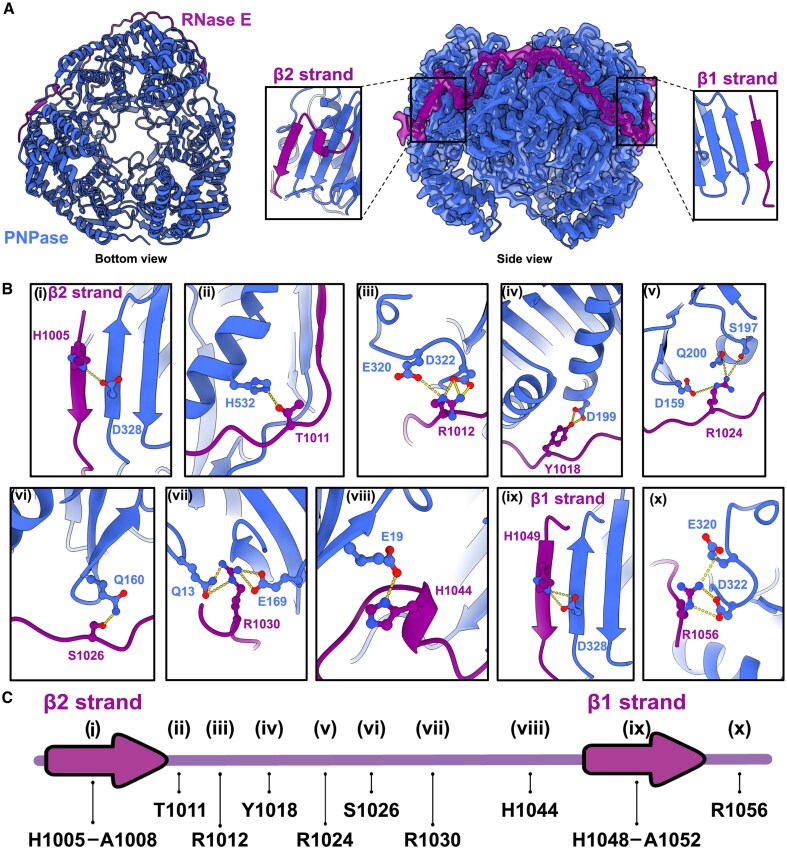
Cryo-EM analysis of the PNPase:RNase E interaction. (**A**) Model of PNPase:RNase E. The PNPase core (1–550) is in blue and the region of RNase E extending from residue 1005 – 1061 is in purple. The two β-strands are labelled as β1 strand and β2 strand. On the right-side of the panel, the Cryo-EM map is coloured according to the atomic model presented in panel A. PNPase core is in blue, and the RNase E peptide is coloured in purple. Cryo-EM reveals that one RNase E protomer can interact with two adjacent PNPase protomers. The two panels highlight the two β-strands, which are structurally similar. (**B**) Residues of RNase E (purple) interacting with PNPase (blue). (**C**) Schematic of the RNase E peptide modelled by cryo-EM and identified as the extended PNPase binding site. The interactions described in panel A are mapped back to the scheme with the respective residue highlighted. The β-strands are represented by arrows.

To distinguish the peptide termini and to break the symmetry imposed by the trimeric PNPase core, a monomeric ultra-stable GFP tag (muGFP) was fused to the C-terminal end of the RNase E peptide 921–1061. Purified RNase E 921–1061-muGFP peptide was combined in molar excess with the PNPase core, and the complex structurally characterized by cryo-EM. A graphical summary of the cryo-EM data processing is represented in Supplementary Fig. S2. 2D class averages show diffuse density adjacent to the PNPase core that corresponds to the muGFP tag, as confirmed by an initial 3D reconstruction at low resolution (Supplementary Fig. S1). Several rounds of focused 3D-classification and focused 3D-refinement resulted in a cryo-EM map at overall 2.6 Å resolution (Fig. 3A), allowing an atomic model to be built for the PNPase core:RNase E complex using the automated model building tool ModelAngelo [31] (Fig. 3A). The cryo-EM map is well-defined for the RNase E peptide from residue 1003 to residue 1057, therefore, only these residues were built in the atomic model. The model confirms the presence of the β-strand between residues 1048–1053 (β1 strand), and a second β-strand was identified and built, formed by the strongly conserved residues 1005 – 1008 (β2 strand), and similar to the residues forming β1 strand (Fig. 3A). 3D classification and 3D refinement confirmed that only one RNase E peptide engages with a PNPase trimer, interacting with two protomers (Fig. 3A), leaving the third ribonuclease protomer exposed and unoccupied. The binding mode, where one RNase E CTD engages one PNPase trimer, is consistent with mass estimates measured by size-exclusion chromatography coupled with multi-angle laser light scattering (SEC-MALLS; Supplementary Table S2). From the cryo-EM map, no density can be seen corresponding to residues 921–1000, suggesting these residues do not interact with PNPase, or the interaction is not sufficiently stable to be visualized by cryo-EM.

The structural model identifies several residues of RNase E interacting with PNPase. First, the β-strands resemble one another both in length, sequence and interaction pattern: His1005 on β2 strand and His1049 on β1 strand interact with the Asp328 present on the β-sheet of one PNPase protomer (Fig. 3Bi, Bix). Preceding β1 strand, His 1044, part of the pseudo-helix, forms an interaction with Glu19 on one PNPase protomer (Fig. 3Bviii). Following β1 strand, some residues of the Pro-rich motif spanning from residues 1053–1061 could be modelled in the cryo-EM map. In particular, Arg1056 form polar interactions with PNPase residues, namely Glu322 and Asp320 (Fig. 3Bx). The strong interaction provided by Arg1056 explains the decrease in binding affinity for the RNase E peptide 985–1053, which lacks this residue (Fig. 2E and F, and Table 1). Overall, only three residues from the previously characterized binding site, from residues 1039 to 1061 [19], form interactions with the PNPase protomer. On the other hand, the atomic model presented here reveals that the upstream region, from residues 1005 to 1039, forms several interactions with the two PNPase protomers engaged by RNase E peptide, which are not limited to the residues forming the β-strand or the proline motif. Following the β-strand formed by residues 1005–1008, β2 strand, the proline motif expanding from residues 1009–1017 forms several interactions with PNPase: Thr1011 interacts with the exposed His522 on the α-helix of PNPase (Fig. 3Bii), and this interaction is followed by Arg1012, which forms three salt bridges with Asp320 and Glu322 (Fig. 3Biii), resembling the interaction formed by Arg1056. Moreover, following the Pro-rich motif, several other residues form interactions with PNPase, such as Tyr1018 interacting with Glu199 (Fig. 3Biv), Arg1024, which interacts with Ser197 (Fig. 3Bv), Ser1026 interacting with Gln160 (Fig. 3Bvi) and finally Arg1030 interacting with Gln13 and E169 (Fig. 3Bvii).

Overall, our cryo-EM data provide insights into an extended binding site for PNPase on RNase E, from residue 1005–1061. This site is composed of two short regions forming β-stands upon interaction with the β-sheet exposed on the PNPase core and are followed by two proline motifs punctuated with charged residues. The residues between the two β-stands form several interactions with the PNPase protomer engaged in the interaction with RNase E. The numerous interaction points between PNPase and RNase E 1005–1039 (Fig. 3B and C), explains why the binding affinity of the extended binding site is boosted nearly three orders of magnitude compared to the peptide 1039–1061 (Table 1). Each Arg in the two proline motifs, namely Arg1056 from the β1 motif (1053–1061) and Arg1012 from the β2 motif (1009–1017) form three salt bridges with different residues on PNPase, suggesting that Arg1012 and Arg1056 are key residues for the interaction with PNPase.

To directly test the effect of Arg1012 and Arg1056 on the binding of RNase E to PNPase, the binding to PNPase of different RNase E peptides carrying point mutations of these residues to alanine (namely R1012A, R1056A, and R1012AR1056A) was tested and measured by SPR (Supplementary Fig. S3 and Supplementary Table S3). The mutation of Arg1012 does not significantly affect the K_D_ of the interaction and the association rate but shows a faster dissociation rate compared to the wildtype (Supplementary Fig. S3 and Supplementary Table S3). For the Arg1056 mutation, the association rate is ten-fold slower, and the *K*_D_ decreases roughly 13-fold compared to the wildtype (Supplementary Fig. S3 and Table S3). This result shows that Arg1056 has a stronger impact on the binding to PNPase compared to Arg1012. This is in line with SPR measurements for binding of RNase E 985–1053, lacking Arg1056, which has significantly weaker binding compared to the RNase E peptide including the region with Arg1056 (Fig. 2 and Table 1). Finally, the mutation of both Arg1012 and Arg1056 significantly affected the binding to PNPase, resulting in a *K*_D_ in the low micromolar range (Supplementary Fig. S3, Supplementary Table S3). These results corroborate that both residues, Arg1012 and Arg1056, are important for the interaction of RNase E with PNPase, and these two residues affect the interaction in different ways. Notably, the association rate for the double mutant is about two orders of magnitude slower compared to the wild type peptide, and ten-fold slower than the slowest-binding mutant (R1056A). This observation suggests a strong synergy of the two arginines in the detailed mechanism for initial encounter and subsequent accommodation of the peptide and PNPase.

### Effects of the RNase E-PNPase interaction on RNA binding and cleavage

The cryo-EM map indicates that RNase E interacts with the PNPase core, but not with the KH and S1 RNA-binding domains (Supplementary Fig. S1). While the interaction with the RNase E C-terminal segment is not anticipated to affect PNPase RNA-binding, it may impact catalytic activity by constraining movements of PNPase catalytic core associated with substrate interaction [36]. Moreover, in *C. crescentus* the interaction of PNPase with RNase E CTD leads to its recruitment into RNase E-containing biomolecular condensates which stimulates PNPase’s catalytic activity [4].

To explore the functional effect of the interaction with RNase E on the catalytic activity of PNPase, we compared the catalytic activity of PNPase in the presence and absence of the RNase E peptide 1000–1061, using RNAs known to be substrates of PNPase, such as RyhB, CyaR, GcvB, and MicA (Supplementary Fig. S4). The *in vitro* assays show that the phosphorolytic activity of PNPase on RyhB and CyaR is boosted in the presence of RNase E (1000–1061) (Supplementary Fig. S4A and B). However, PNPase activity on MicA and GcvB did not show significant difference with the presence or absence of the RNase E peptide. These results suggest that the interaction of the ribonucleases could also enhance the catalytic activity of the *E. coli* PNPase in the context of the degradosome assembly for some RNA targets.

### Interaction of RNase E and PNPase in other bacteria

**Figure 4.**
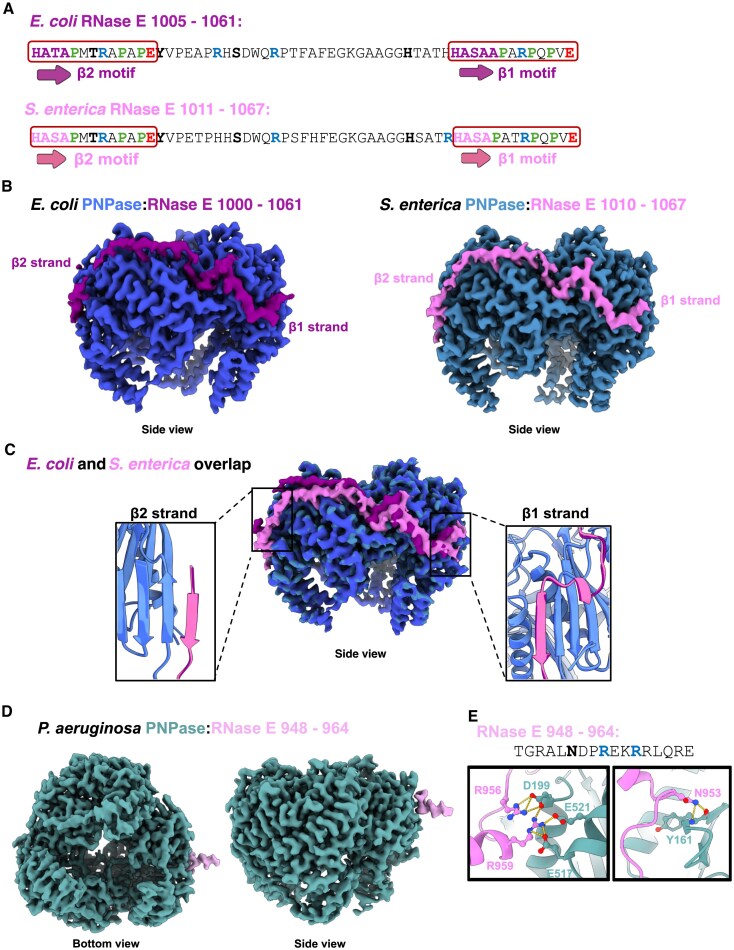
PNPase and RNase E have different modes of interaction in divergent bacteria. (**A**) Sequence comparison of the PNPase binding site in *E. coli* and *S. enterica* RNase E. These two bacteria are relatively close in evolution and have a high percentage of sequence identity. The β-strands are labelled as β1 and β2 and are in red rectangles. (**B**) Comparison of cryo-EM structures for the PNPase:RNase E complexes in *E. coli* and *S. enterica*. (**C**) Overlap of the *E. coli* and *S. enterica* structures shown in panels (A) and (B). The two insets show the overlap of two β-strands, β1 and β2. The same interaction mode is observed, where one RNase E peptide (purple in *E. coli* and pink in *S. enterica*) interacts with two PNPase protomers (electric blue in *E. coli* and steel blue in *S. enterica*). (**D**) The cryo-EM map for RNase E and PNPase from *P. aeruginosa* shows a different interaction mode where a short motif on RNase E (pink) presents an α-helix to interact with one PNPase protomer (steel). (**E**) Example of interactions between RNase E and PNPase in *P. aeruginosa*. RNase E residues are coloured in pink, and PNPase residues are coloured in steel.

*Salmonella enterica* and *E. coli* are closely related species that present strong sequence conservation both for PNPase and RNase E (Figs 2B and 4A). Like *E. coli*, *S. enterica* presents two proline-rich motifs, which are signatures of the avid PNPase binding site. We used cryo-EM to investigate the interaction between RNase E and PNPase in *S. enterica* serovar Typhimurium (for simplicity, hereafter, referred to as *S. enterica*). The complex PNPase core: RNase E 921–1067-muGFP from *S. enterica* was assembled following the same approach used for the *E. coli* complex and analysed by cryo-EM, generating a 2.42 Å resolution reconstruction showing clear density for an RNase E peptide spanning residues 1010–1064 and interacting with two PNPase protomers through two β-strands and charged residues, as seen for the *E. coli* enzyme (Fig. 4B and C). This result from *S. enterica* enzymes corroborates the identification of an extended binding motif for PNPase in closely related species.

The interaction between PNPase and RNase E in the pathogen *P. aeruginosa* has been reported to contribute to infective virulence and other complex phenotypes [37]. *E. coli* and *P. aeruginosa* are distant bacterial lineages, and their RNase E sequences differ significantly for the intrinsically disordered region and for the region corresponding to the identified binding sites for PNPase [37]. To structurally investigate the interaction, the complex between the PNPase core and an MBP-tagged version of the C-terminal end of RNase E (883 – 1057) from *P. aeruginosa* was assembled and analysed by cryo-EM. The map reveals a new mode of interaction between the two ribonucleases, where a region of the intrinsically disordered part of RNase E from residues 948–964 interacts with the RNase PH domain of PNPase by the formation of an α-helix (Fig. 4D). The interaction is mediated by charged residues forming hydrogen bonds and salt bridges (Fig. 4E). Residues of the α-helix form several interactions with the backbone of the PNPase protomer, and one example is shown in the right panel of Fig. 4E. Moreover, two Arg residues form several salt bridges with negatively charged residues on the PNPase (left panel, Fig. 4E). Although the interacting area is smaller compared to what was found for *E. coli* and *S. enterica*, the number of interactions provides sufficient stability to be visualized by cryo-EM. Only a few residues forming the α-helix could be modelled due to poor resolution, suggesting high flexibility. The mode of interaction agrees with predictions from AlphaFold3 [38] (not shown).

Taken together, these different modes of interaction, summarized in Fig. 4, represent examples where distant bacterial lineages evolved different means of recruiting PNPase to RNase E within the RNA degradosome. The common requirement for association of PNPase with the intrinsically disordered portion of RNase E indicates the importance of the interaction and its capacity to flexibly manoeuvre.

## Discussion

The multi-enzyme RNA degradosome has presented long-standing puzzles regarding its compositional stoichiometry, organization and propensity to coalesce *in vivo*. The degradosome is highly dynamic, enriched in intrinsically disordered domains, and has variable composition depending on environmental conditions. The canonical components appear to be stably associated, namely enolase, RhlB helicase and PNPase. Other partners—such as sRNA/Hfq complexes—interact fleetingly. One question in understanding the dynamic and constitutive organization of the degradosome is the interaction of the canonical and variable components within the complex.

In this study, we found evidence for an extended PNPase binding site at the C-terminal end of RNase E that can mediate strong, cooperative interaction with PNPase. The RNase E-PNPase interaction in *E. coli* uses a recurrent mode of molecular recognition, common to many different complexes, through the combination of an exterior exposed strand of a β-sheet and a partner with microstructural motif of a β-strand (β-augmentation), capped with a proline element (Fig. 3). Binding measurements revealed that the proline region and the very C-terminal end of the binding site for PNPase are required for an avid interaction (Table 1). The weak interaction between residues 1039–1061 of RNase E with PNPase helps to pre-organize the upstream binding motif of RNase for interaction with PNPase, forming several interactions up to the formation of the second β-strand (Fig. 3) and conferring strong overall affinity for this enzyme. Moreover, structural analysis of the interaction showed that charged residues present in these proline regions, such as Arg1056 or Arg1012, provide an anchor to PNPase, suggesting a signature element of the PNPase binding motif (Fig. 3 and Supplementary Fig. S3).

Cryo-EM confirms that only one RNase E monomer interacts with one PNPase trimer with the formation of two extended β-sheet interactions and tracking of an extended peptide along a surface groove (Fig. 3). The stoichiometry of the interaction between the two ribonucleases, where one RNase E monomer interacts with one PNPase trimer, agrees with previous biochemical studies of the degradosome [39]. Moreover, SEC-MALLS analysis corroborates the binding mode of one RNase E CTD to one PNPase trimer, even in the presence of the other degradosome components, such as RhlB and enolase (Supplementary Table S2). The proposed mode of interaction leaves exposed a potential binding site in one protomer in the PNPase trimer (Fig. 3), which opens the possibility of a hypothetical additional recognition segment residing in RNase E. Along the entire C-terminal tail of RNase E, other regions have similarity to the PNPase binding motif and show sequence conservation in bacteria (Supplementary Fig. S6). These regions do not have an assigned function or role, and the conservation suggests they may carry either a functional or structural role. Considering the interaction of the two ribonucleases in the context of the degradosome assembly, the intrinsically disordered nature of the C-terminal portion of RNase E may allow PNPase to interact with the putative additional binding site, to come into transient proximity with the catalytic site of RNase E (Supplementary Fig. S6B), and this fleeting interaction is envisaged to impact on its catalytic action in several ways. First, the proximity could boost mRNA decay efficiency, as RNase E cleavage of mRNAs produces RNA fragments with unprotected 3′ end, substrates of the locally tethered PNPase. Moreover, PNPase can act in a protective mode as part of an “RNA carrier” complex formed with Hfq and sRNAs [15], and recent cryo-EM studies of the RNA carrier complex formed by PNPase:Hfq:sRNA show that Hfq cooperates with the KH and S1 domains of PNPase to capture the RNA, and this interaction with Hfq prevents the 3′ end of the sRNA from entering the central channel, protecting it from degradation [17]. The proximity of PNPase and RNase E catalytic domain could, in principle, facilitate substrate handover from the RNA-binding element and from the PNPase/Hfq/sRNA/mRNA complex to RNase E [10, 16, 17].

The interaction between RNase E and PNPase has been conserved in many bacterial lineages. Regardless, distinct interaction modes have evolved to ensure tight interaction of the two ribonucleases in the cell. Previous studies showed how in *Caulobacter crescentus*, PNPase binds a short segment at the C-terminal end of RNase E, containing a GWW motif found to be strongly conserved in α-proteobacteria. Crystallography studies reveal that the GWW motif occupies a hydrophobic pocket in the PNPase core, which is spatially distant (∼60 Å) from interaction site seen for the corresponding bind sites of the *E. coli* and *S. enterica* enzymes [36]. In cyanobacteria, PNPase interacts with a site in the RNase E CTD bearing a nonapeptide with consensus sequence RRRRRRSSA [40]. In this study we show how *E. coli* and *S. enterica*, two closely related bacterial species, have the same structural pattern of interaction with two adjacent PNPase protomers, leaving the third protomer exposed (Fig. 4). We also structurally characterized the interaction between RNase E and PNPase in *P. aeruginosa*, revealing yet another mode of interaction involving the docking of the end of a long helix from RNase E to the surface of a PNPase protomer (Fig. 4). Overall, these structures represent an example where different structural motifs are used to achieve the interaction of the two ribonucleases. While the details of the molecular recognition differ, the common occurrence of interaction between RNase E and PNPase, two key enzymes of bacterial RNA metabolism, support the hypothesis that the physical interaction provides functional benefit to diverse bacterial organisms.

## Supplementary Material

gkaf960_Supplemental_File

## Data Availability

The maps and models have been deposited with the PDB and cryoEM codes 9QH0/EMD-53151 for *E. coli* polynucleotide phosphorylase in complex with recognition site of RNase E; 9QH3/EMD-53153 for *P. aeruginosa* polynucleotide phosphorylase in complex with recognition site of RNase E; and 9IBH/EMD-52810 for *Salmonella typhimurium* polynucleotide phosphorylase in complex with recognition site of RNase E.
