# Supplementary material for: A multi-dentate, cooperative interaction between endo- and exo-ribonucleases within the bacterial RNA degradosome
Source: Nucleic Acids Res. 2025 Oct 2;53(18):gkaf960. doi: 10.1093/nar/gkaf960 (PMC12489471; doi:10.1093/nar/gkaf960)
Supplement: gkaf960_Supplemental_File [file gkaf960_supplemental_file.pdf]

Supplementary Figures and Tables

A multi-dentate, cooperative interaction between endo- and exo-ribonucleases within the bacterial RNA degradosome

Giulia Paris, Kai Katsuya-Gaviria, Hannah Clarke, Margaret Johncock, Tom Dendooven, Aleksei Lulla* & Ben F. Luisi*

Supplementary Figure 1. Cryo-EM of PNPase:RNase E 960–1061 complex in *E. coli*

Supplementary Figure 2. Cryo-EM data processing workflow for PNPase core:RNase E 921–1061 – muGFP from *E. coli*.

Supplementary Figure 3. Mutations of key RNase E residues on the PNPase binding sites affect binding affinity and kinetics.

Supplementary Figure 4. PNPase catalytic activity is boosted for some RNAs when bound to RNase E.

Supplementary Figure 5. FSC curves of the three datasets.

Supplementary Figure 6. Weaker, transient interactions may occur at different sites on RNase E, bringing the two ribonucleases in close contact.

Supplementary Table S1. RNase E peptides prepared in this study.

Supplementary Table S2. SEC-MALLS molecular masses.

Supplementary Table S3. SPR analysis of RNase E mutants.

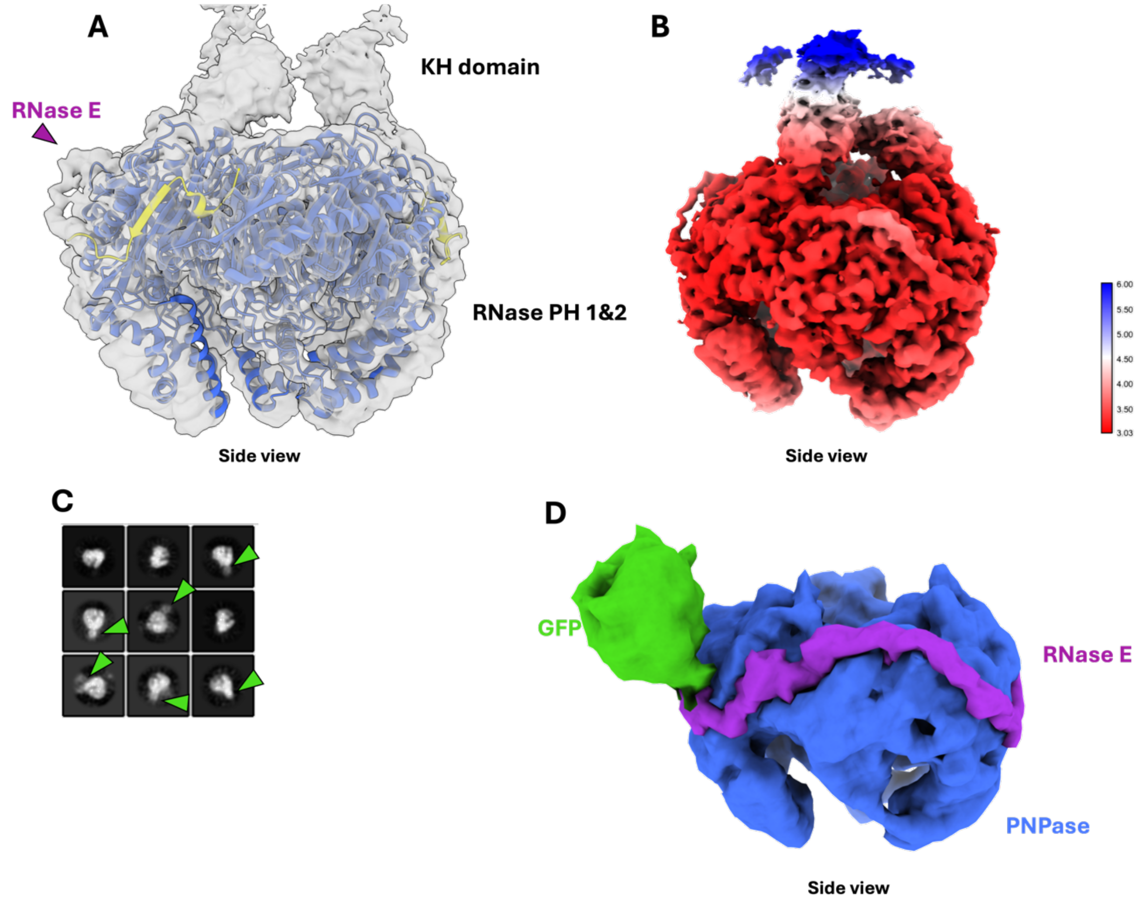

Supplementary Figure 1. Cryo-EM of PNPase:RNase E 960–1061 complex in *E. coli* A) 3D volume reconstruction of PNPase:RNase E 960–1061. As seen in previous studies, the KH and S1 domains are highly flexible and therefore not well resolved (17). The map shows density for the KH domains, where helices can be distinguished. The density for the S1 domains instead is poor and therefore was excluded from the image. The PNPase core is well resolved, and the previously described X-ray structure (PDB:3GCM) (19) has been manually docked in to identify the RNase E peptide bound to PNPase. The PNPase core is coloured in blue, and the RNase E peptide described by X-ray (19) is in yellow. Extra-density corresponding to RNase E 960 – 1061 can be seen interacting with the PNPase core (purple arrows), bridging two β-strands. **B)** Cryo-EM map of PNPase:RNase E 960 -1061 colour-coded by local resolution. **C)** Selected 2D class averages for the PNP:RNase E – GFP complex. The lime green triangles point to the density corresponding to the GFP tag. The 2D class averages show that only one GFP tag is bound to PNPase. **B)** Initial map generated from the 2D class average, false colour-coded for the components. An AlphaFold3 prediction (45) of the PNPase core:RNase E 1000 – 1061 – GFP complex was manually docked into the map, which is coloured as follows: PNPase core (blue) is shown to interact with one RNase E – GFP (purple and green, respectively). Only one RNase E peptide interacts with two PNPase protomers, leaving the third binding site empty.

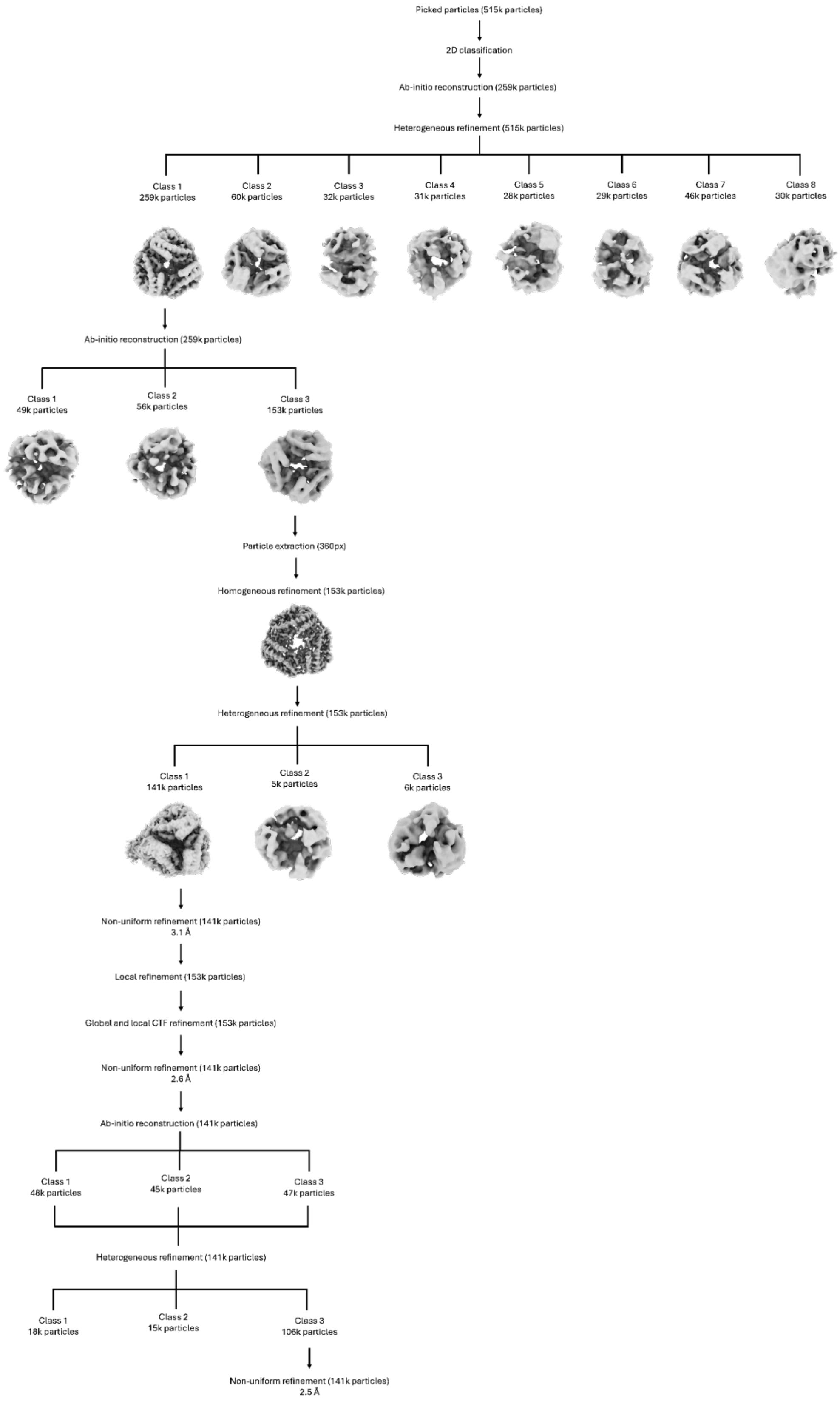

Supplementary Figure 2. Cryo-EM data processing workflow for PNPase core:RNase E 921–1061 – muGFP from *E. coli*. Workflow of cryo-EM data processing from particle picking to the generation of the final map used for model building. All processing steps were performed in cryoSPARC (40).

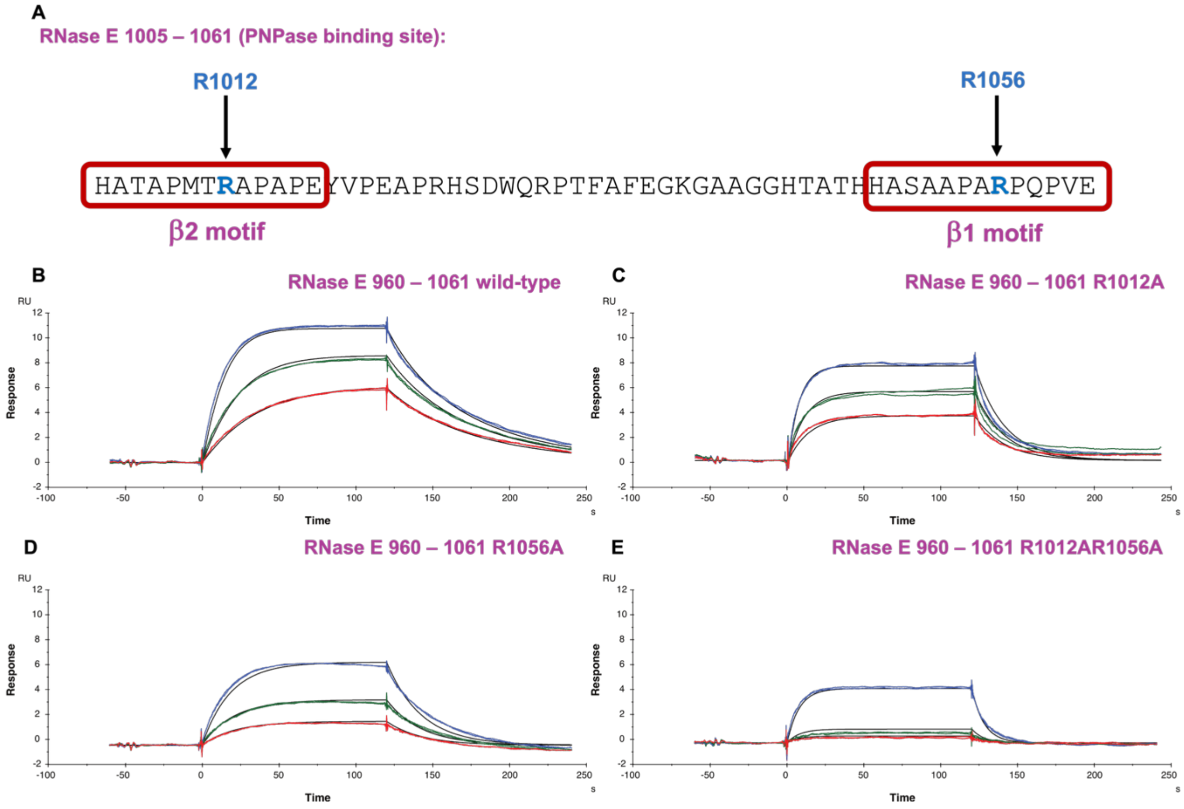

Supplementary Figure 3. Mutations of key RNase E residues on the PNPase binding sites affect binding affinity and kinetics. A) Schematic of the PNPase binding site (RNase E 1005 - 1061). The β1 and β2 motifs are highlighted by the red box. The Arginines of interest are coloured in blue. SPR profiles of the R1012A mutant **(C)**, the R1056A mutant **(D)** and the double point mutant R1012AR1056A **(E)** show differences in the kinetics compared to the wildtype RNase E 960 – 1061 **(B)**. R1012A and R1056A show a faster off-rate compared to the wildtype, suggesting a weaker binding. The double mutant shows a significant decrease in binding, and a faster off-rate compared both to the wildtype and the single point mutants.

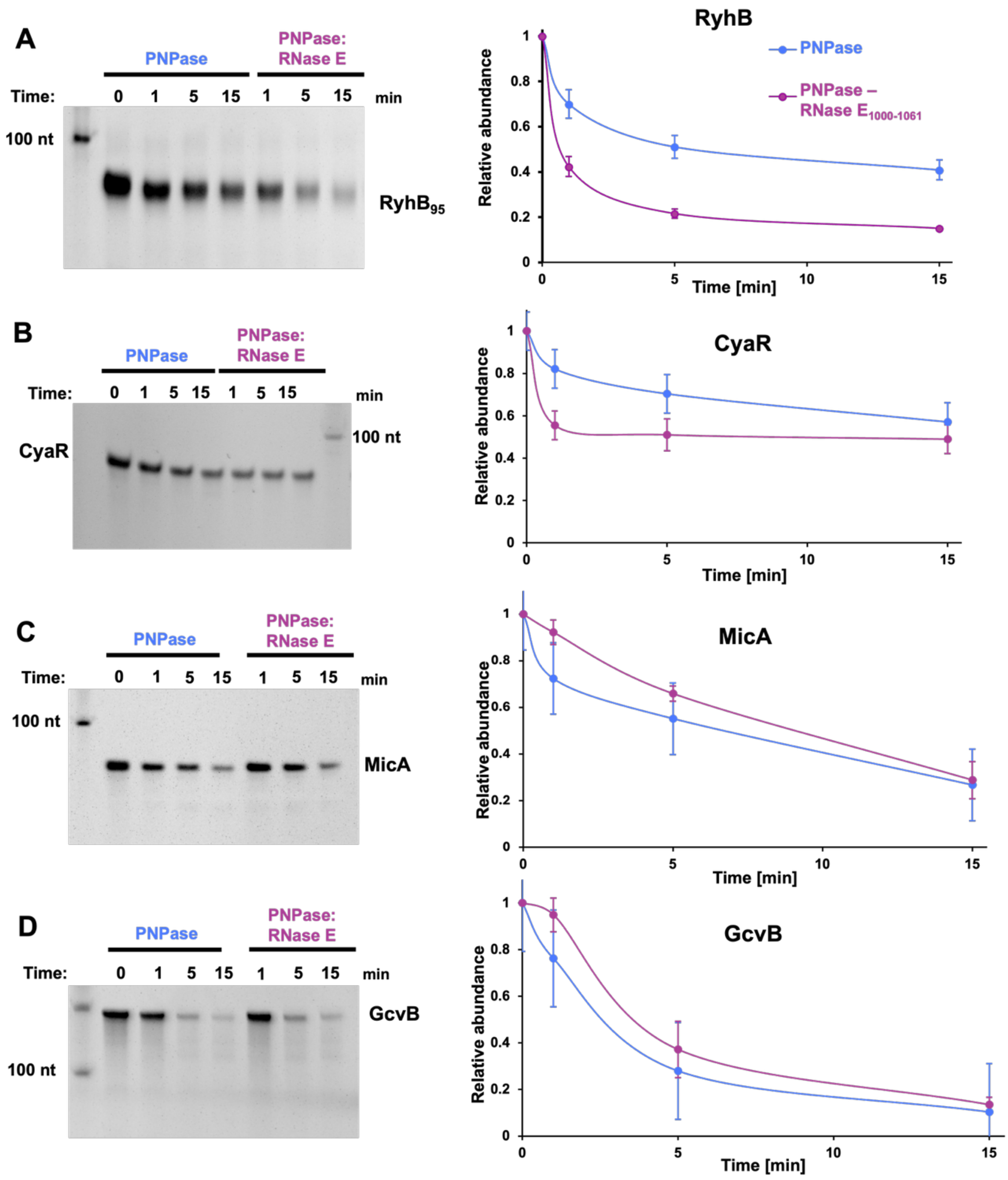

Supplementary Figure 4. PNPase catalytic activity is boosted for some RNAs when bound to RNase E. Time course reactions of degradation of different RNAs at 500 nM, namely RyhB in panel **A**, CyaR in panel **B**, MicA in panel **C** and GcvB in panel **D**, with 50 nM PNPase free or bound to RNase E (1000 – 1061). The reactions were stopped at the indicated time points (1, 5, 15 min). Time point 0 was taken immediately before enzyme addition. On the right side of the figure, relative abundances of the different RNAs over time from experiments on the left. Data are mean ± SD from three independent reactions. PNPase shows a more efficient cleavage for some RNAs, like RhyB **(A)** and CyaR **(B)**, when bound to the RNase E peptide 1000 – 1061. For other RNAs, such as MicA **(C)** and GcvB **(D)**, the cleavage efficiency of PNPase is the not significantly different in the presence or absence of the RNase E peptide.

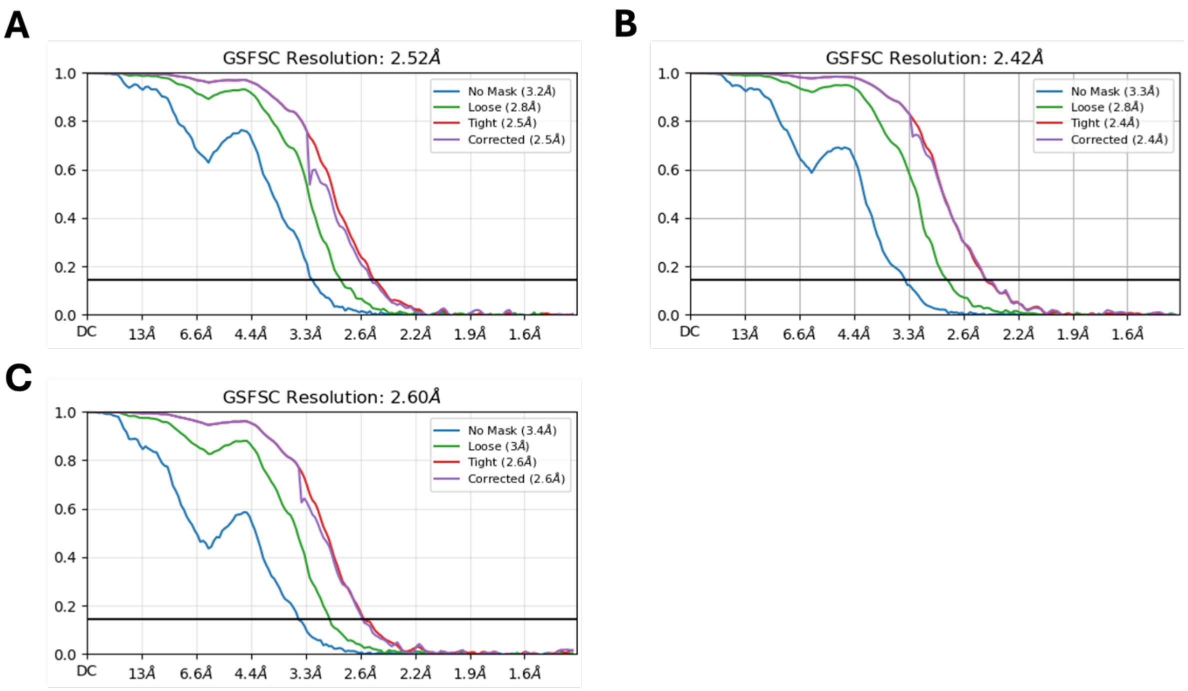

Supplementary Figure 5. FSC curves of the three datasets. A) FSC curve of *E. coli* PNPase core:RNase E 921-1061-muGFP. **B)** FSC curve of *S. enterica* PNPase core:RNase E 921-1061-muGFP. **C)** FSC curve of *P. aeruginosa* PNPase core:MBP-RNase E 883-1067.

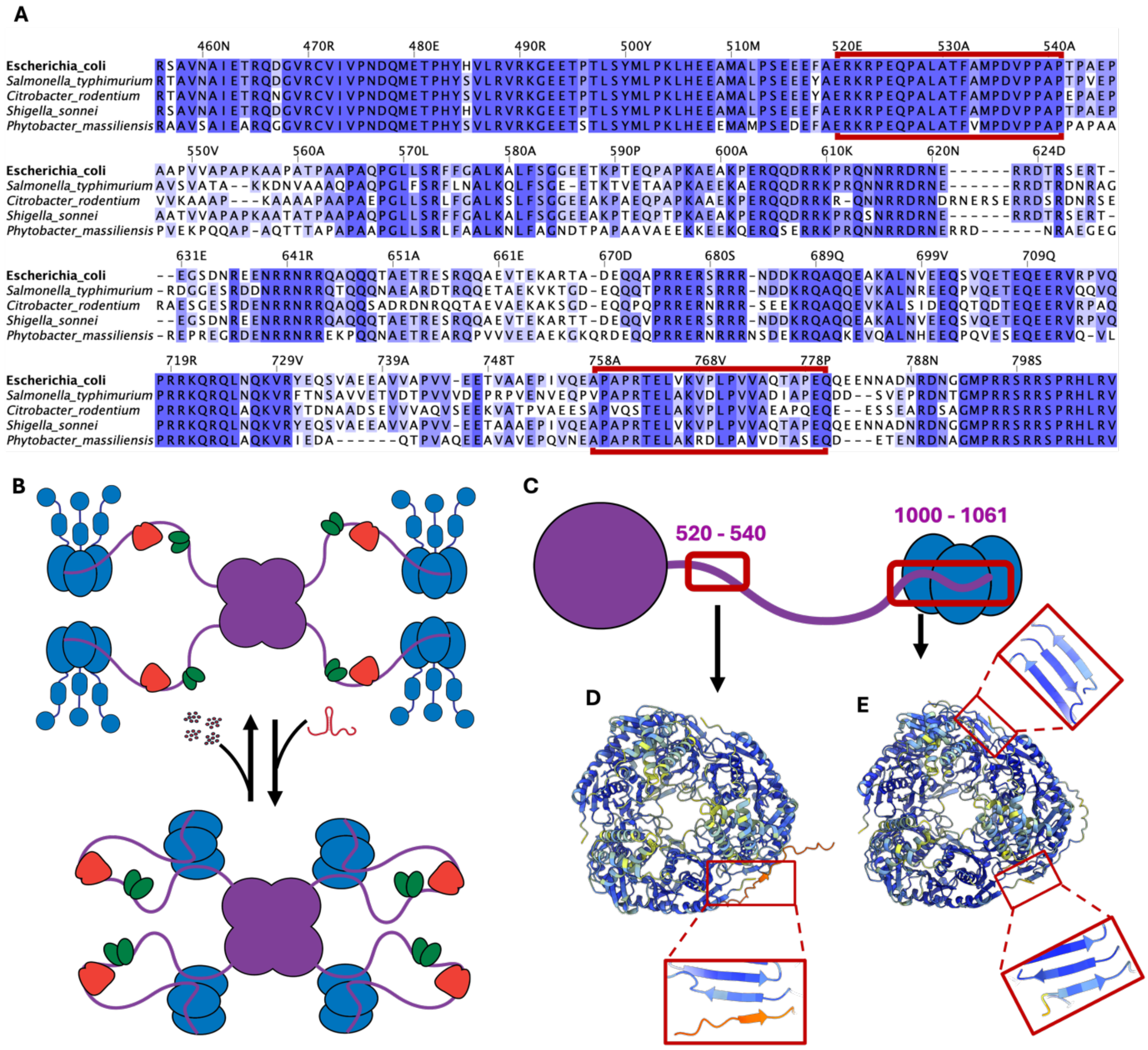

Supplementary Figure 6. Weaker, transient interactions may occur at different sites on RNase E, bringing the two ribonucleases in close contact. **A)** Multisequence Alignment identifies other conserved regions along the intrinsically disordered domain of RNase E, suggesting these regions may have a functional role. The regions of interest (520 – 540, 755 – 778) that show sequence conservation but do not have a known structural or functional role yet are highlighted by red boxes. **B)** Schematic of two possible conformations of the RNA degradosome: an open conformation (on the top), which switches to a closed conformation upon binding to RNA substrates. In the closed conformation (at the bottom) the two ribonucleases are in proximity, assured by the interaction of PNPase with the hypothetical third binding site. **C)** Schematic of one RNase E monomer (purple), where the PNPase binding site (PBS) extending from residues 1000–1061 is highlighted by the red box. RNase E 510–560 has been predicted for binding to PNPase using AlphaFold3 (45) (panel **D**). The known interaction site was also predicted using AlphaFold3, using RNase E 1000 – 1061 (panel **E**). The AlphaFold3 prediction models are coloured according to the respective pIDDT —where blue equals to high confidence and red low confidence (45).

Supplementary Table S1. Peptide segments from the RNase E C-terminal domain used for SPR analysis.

RNase E segment	Length (aa)	MW (kDa)	pI
1022 - 1061	40	4.203	8.81
985 – 1061	77	8.098	5.57
985 - 1053	69	7.223	5.55
960 - 1038	79	8.410	4.43
960 – 1061	102	105.84	4.82
960 – 1061 R012A	102	104.99	4.70
960 – 1061 R1056A	102	104.99	4.70
960 – 1061 R1012AR1056A	102	104.13	4.58

Supplementary Table S2. Molecular masses by Size exclusion chromatography with multi-angle laser light scattering

Sample	Mn number-averaged solute molar mass, Da	Mw weight-averaged solute molar mass, Da	Polydispersity (Mn/Mw)	Expected MW, Da	Difference Mn from expected mass	Buffer<br>A=50 mM Tris pH 7.5, 100 mM NaCl, 100 mM KCl, 0.01% w/v b-DDM, 1 mM TCEP<br>B=A w/o bDDM
E. coli PNPase core, lacking S1 and KH domains	163,300	163,600	1.002	178,782 trimer	8.7%	A
E. coli PNPase	204,600	204,900	1.001	231,302 trimer	11.5%	A
E. coli PNPase, RNase E 960-1061	217,100	218,000	1.004	241,887 (1:1 ratio)	10.2%	A
E. coli PNPase, RNase E 583-1061, avi-tagged	255,700	255,900	1.001	286,845 (1:1 ratio)	10.8%	B
RNase E 583-1061, avi-tagged	52,930	53,310	1.007	55,543	4.7%	B
E. coli PNPase, RNase E 511-1061	264,000	264,600	1.002	292,480 (1:1 ratio)	9.7%	B
RNase E 511-1061	63,870	64,020	1.002	61,178	4.4%	B
Bovine serum albumin	63,250<br>63,300<br>63,900	63,270<br>63,350<br>64,200	1.000<br>1.001<br>1.005	66,430	4.8%<br>4.7%<br>3.8%	A<br>B<br>B

Supplementary Table S3. Rate constants and binding strengths of interactions of RNase E mutants and PNPase. The SPR binding profiles are shown in Supplementary Figure 3.

Peptide	K_a (1/Ms)	K_d (1/s)	K_D (M)
RNase E 960 – 1061 wildtype	$1.5*10^{6}$	0.026	$1.8*10^{-8}$
RNase E 960 – 1061 R1012A	$1.7*10^{6}$	0.052	$3*10^{-8}$
RNase E 960 – 1061 R1056A	$1.5*10^{5}$	0.045	$2.4*10^{-7}$
RNase E 960 – 1061 R1012AR10546A	$1.4*10^{4}$	0.12	$9*10^{-6}$
